# A prospective evaluation of the safety and efficacy of the TAXUS Element paclitaxel-eluting coronary stent system for the treatment of de novo coronary artery lesions: Design and statistical methods of the PERSEUS clinical program

**DOI:** 10.1186/1745-6215-11-1

**Published:** 2010-01-07

**Authors:** Dominic J Allocco, Louis A Cannon, Amy Britt, John E Heil, Andrey Nersesov, Scott Wehrenberg, Keith D Dawkins, Dean J Kereiakes

**Affiliations:** 1 Boston Scientific Corporation, One Boston Scientific Place, Natick, MA 01760, USA; 2 Cardiac and Vascular Research Center of Northern Michigan, Northern Michigan Regional Hospital, Petoskey, 560 W Mitchell St., Suite #480, Petoskey, MI, USA; 3 The Christ Hospital Heart and Vascular Center/The Lindner Center for Research and Education at The Christ Hospital, 2123 Auburn Avenue, Suite 424, Cincinnati, OH, USA

## Abstract

**Background:**

Paclitaxel-eluting stents decrease angiographic and clinical restenosis following percutaneous coronary intervention compared to bare metal stents. TAXUS Element is a third-generation paclitaxel-eluting stent which incorporates a novel, thinner-strut, platinum-enriched metal alloy platform. The stent is intended to have enhanced radiopacity and improved deliverability compared to other paclitaxel-eluting stents. The safety and efficacy of the TAXUS Element stent are being evaluated in the pivotal PERSEUS clinical trials.

**Methods/Design:**

The PERSEUS trials include two parallel studies of the TAXUS Element stent in single, de novo coronary atherosclerotic lesions. The PERSEUS Workhorse study is a prospective, randomized (3:1), single-blind, non-inferiority trial in subjects with lesion length ≤28 mm and vessel diameter ≥2.75 mm to ≤4.0 mm which compares TAXUS Element to the TAXUS Express^2 ^paclitaxel-eluting stent system. The Workhorse study employs a novel Bayesian statistical approach that uses prior information to limit the number of study subjects exposed to the investigational device and thus provide a safer and more efficient analysis of the TAXUS Element stent. PERSEUS Small Vessel is a prospective, single-arm, superiority trial in subjects with lesion length ≤20 mm and vessel diameter ≥2.25 mm to <2.75 mm that compares TAXUS Element with a matched historical bare metal Express stent control.

**Discussion:**

The TAXUS PERSEUS clinical trial program uses a novel statistical approach to evaluate whether design and metal alloy iterations in the TAXUS Element stent platform provide comparable safety and improved procedural performance compared to the previous generation Express stent. PERSEUS trial enrollment is complete and primary endpoint data are expected in 2010. PERSEUS Workhorse and Small Vessel are registered at http://www.clinicaltrials.gov, identification numbers NCT00484315 and NCT00489541.

## Background

Drug-eluting stents, including paclitaxel-eluting stents, have been shown to reduce angiographic restenosis and the need for repeat revascularization following coronary angioplasty compared to bare metal stents [1,2]. However, repeat revascularization is still required in approximately 7-10% of patients (versus 20-25% with bare metal stents) [3]. It has been proposed that the thickness of stent struts may impact the ability of the stent to reduce restenosis. Compared to first generation stents with strut thicknesses of approximately 130-150 μm, stents with thinner stent struts (80-100 μm) have been associated with a lower late luminal loss and less neointimal volume obstruction after stenting, possibly as a result of less stent-induced arterial injury and inflammation [4,5]. Thinner stent struts also facilitate deliverability through tortuous vessel anatomy. However, the development of thinner struts with 316L stainless steel limits both radiographic visualization (ie, radiopacity), which is required to ensure proper stent placement, and the necessary radial strength for adequate stent expansion, particularly in resistant fibrocalcific target lesions [6].

The TAXUS Element paclitaxel-eluting coronary stent uses the same polymer and has similar paclitaxel release kinetics as the earlier TAXUS Express [1,7,8] and TAXUS Liberté [2,9-11], 9-11 316L stainless steel stent systems, but employs a new 81 μm platinum chromium alloy in a design intended to improve deliverability, increase radiopacity, and maintain low stent recoil when compared with previous TAXUS stent designs. The PERSEUS program evaluates the TAXUS Element stent for the treatment of single de novo atherosclerotic lesions using a novel Bayesian statistical approach to increase efficiency.

## Methods/Design

### Device Description

The TAXUS Element stent is a novel, balloon-expandable, 81 μm, platinum chromium alloy stent pre-mounted on a high-pressure delivery balloon. The pharmacological agent, paclitaxel, is incorporated into a triblock polymer matrix and applied to the surface of the stent to provide controlled release of available paclitaxel (see Appendix A for a detailed description of TAXUS Element and comparison to previous platforms).

### Study Designs

**Figure 1:**
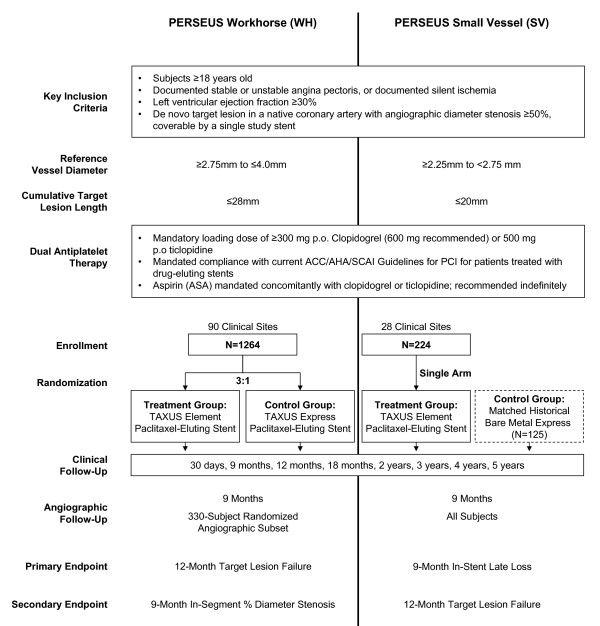
**PERSEUS WH and PERSEUS SV Study Schematic**.

The TAXUS PERSEUS Clinical Trial Program evaluates the TAXUS Element paclitaxel-eluting stent system for the treatment of single, de novo atherosclerotic lesions in two parallel studies (Figure 1).

#### The PERSEUS Workhorse (WH) trial

PERSEUS WH is a prospective, randomized, single-blind, non-inferiority trial which employs a 3:1 randomization to the TAXUS Element or the TAXUS Express paclitaxel-eluting stents respectively. Subjects with target lesion length ≤28 mm and reference vessel diameter (RVD) ≥2.75 mm to ≤4.0 mm were considered for enrollment. Additional inclusion and exclusion criteria are given in Appendix B. Subjects were randomized after successful predilatation of the target lesion and were considered to be enrolled at the time of randomization. The randomization schedules were computer-generated using a pseudo-random number generator and stratified both by clinical site and by the presence or absence of medically treated diabetes. The number of diabetic subjects was capped at 350. In total, 1264 subjects were enrolled at 90 clinical sites in the United States, Australia, New Zealand, and Singapore (see Acknowledgements), of whom 330 subjects were randomly assigned to protocol-mandated 9-month angiographic follow-up (angiographic subset). The primary endpoint is the rate of target lesion failure (TLF) at 12 months post-index procedure. In-segment percent diameter stenosis at 9 months post-index procedure as measured by quantitative coronary angiography (QCA) is the secondary endpoint. Additional clinical and angiographic endpoints in both the WH and Small Vessel studies include target vessel revascularization (TVR), major adverse cardiac events (MACE), stent thrombosis, and technical and procedural success, as well as angiographic late loss and binary restenosis.

#### PERSEUS Small Vessel (SV) Trial

PERSEUS SV is a prospective, single-arm, superiority trial that compares the TAXUS Element stent to a matched bare metal (Express) historical control group garnered from the TAXUS V trial. The control group comprised 125 intent-to-treat subjects with RVD ≥2.25 to <2.75 mm and lesion length ≤20 mm of whom 108 subjects had QCA at 9-month follow-up. A total of 224 subjects from 28 United States sites were enrolled. All subjects in PERSEUS SV are required to undergo a 9-month angiographic assessment. The primary endpoint is in-stent late loss by QCA on 9-month angiographic follow-up and the key secondary endpoint is TLF at 12 months. Additional clinical and angiographic endpoints are similar to the PERSEUS WH study as noted above.

### Endpoint Definitions

TLF is defined as any ischemia-driven revascularization of the target lesion (TLR), myocardial infarction (MI; both Q-wave and non-Q-wave) related to the target vessel, or cardiac death related to the target vessel. If relationship to the target vessel could not be determined with certainty, the event was assumed to be related to the target vessel. MACE is defined as MI, TVR, or cardiac death. Stent thrombosis is defined per historical Boston Scientific Corporation protocol definitions (see Appendix C) and per the Academic Research Consortium definition [12]. Additional clinical and angiographic endpoint definitions are given in Appendix C.

### Follow-up Schedule

For both studies, clinical endpoint measurements were conducted in-hospital and at 30 days, and are planned at 9 months, 12 months, 18 months, 2 years, 3 years, 4 years, and 5 years. Angiographic follow-up at 9 months is planned for subjects randomized to the angiographic subset in the PERSEUS WH study and for all subjects in PERSEUS SV. Starting with the 18-month visit, follow-up will be limited to those study subjects who actually received a study stent (TAXUS Element or TAXUS Express).

### Antiplatelet and Other Concomitant Medical Therapy

Treatment with aspirin and clopidogrel (or ticlopidine) is required for both PERSEUS studies in compliance with the ACC/AHA/SCAI Guidelines for percutaneous coronary intervention (PCI) [13]. Aspirin ≥300 mg was administered orally at least 1 hour prior to catheterization and a clopidogrel oral loading dose of ≥300 to 600 mg was administered (preferably ≥6 hours prior to the procedure, but no later than 2 hours after completion of the index procedure). During the procedure, unfractionated heparin was recommended as necessary to maintain an activated clotting time ≥250 seconds. Alternatively, enoxaparin, bivalirudin or other procedural antithrombotics could be administered per local standard of practice. Abciximab, eptifibatide, and tirofiban could be administered at the discretion of the investigator. Clopidogrel at 75 mg orally daily was required for at least 6 months and ideally up to 12 months in subjects not at high risk of bleeding consistent with the ACC/AHA/SCAI Guidelines for PCI in effect at the time study enrollment began. Five months after the start of study enrollment, the PCI guidelines were revised to recommend 12 months of clopidogrel therapy in all patients receiving drug-eluting stents [13]. In case of allergy or intolerance to clopidogrel, ticlopidine 250 mg orally twice daily was prescribed. Daily aspirin therapy was mandated concomitantly with clopidogrel or ticlopidine and continued indefinitely.

### Criteria for Multiple and Staged Interventions

Only one target lesion segment, treatable by a single stent, was to be considered the target lesion. If separate lesions in 2 different native coronary arteries were eligible, the operator was to decide which lesion would be treated as the target lesion prior to treating any lesion(s) or vessel(s). The assumed culprit lesion was selected as the target lesion (defined as the lesion most likely responsible for a clinical event based on evidence of ischemia or the lesion with greatest percent diameter stenosis on visual estimate). Treatment of one lesion in a single non-target vessel during the index procedure was allowed by protocol prior to treatment of the target lesion. Treatment of the non-study lesion could not require additional unplanned stents, and must have been successful angiographically (see Appendix C) for the subject to be eligible for enrollment into the study. Staged PCI or subsequent planned coronary artery bypass graft procedures were not allowed post-index procedure.

### Angiographic Follow-up

Angiographic follow-up is required at 9 months in the angiographic subset of PERSEUS WH and in all PERSEUS SV subjects. Central analysis of all angiographic studies will be performed by an Angiographic Core Laboratory (Beth Israel Deaconess Medical Center, Boston, MA) using standard qualitative morphologic criteria [14] identical to those used in the TAXUS Express and TAXUS Liberté clinical trials [15].

### Statistical Analysis Plan for the Primary Endpoint

#### PERSEUS WH

For the primary endpoint analysis, Bayesian hierarchical modeling will be used to determine if the 12-month TLF rate for the TAXUS Element stent is non-inferior to the 12-month TLF rate for the TAXUS Express^2 ^paclitaxel-eluting stent system. The hierarchical model will be used to estimate the difference in the 12-month TLF rate between the TAXUS Element and TAXUS Express devices. This hierarchical model involves the TLF rates observed for TAXUS Element and TAXUS Express in patients enrolled in PERSEUS WH, as well as rates observed in data conditionally borrowed from TAXUS IV and V patients (under the Bayesian framework, as described below).

Bayesian methods differ from the more conventional frequentist methods in that they can utilize prior information, potentially increasing the precision of analyses [16]. While frequentist methods also use prior data in trial planning for sample size and power calculations, the evidence for or against the null hypothesis comes solely from the current trial. By utilizing the prior information in assessing trial endpoints, the Bayesian approach may allow for a smaller sample size, thereby minimizing the number of patients exposed to an investigational drug or device during its evaluation. Bayesian analyses may be interpreted in a more intuitive way than frequentist analyses because they treat the parameter of interest as a random variable rather than as a fixed unknown value. Specifically, Bayesian methods provide a posterior probability that a statement is true or false given the prior information and the observed data, whereas the frequentist *P *value provides the probability of observing data as or more extreme than that observed assuming the null hypothesis is true. The Bayesian approach has been supported by the US Food and Drug Administration's (FDA) Center for Devices and Radiological Health for medical device clinical trials when the prior data utilized come from robust clinical studies [17,18]. Bayesian methods were chosen for PERSEUS WH in order to utilize extensive prior data on the TAXUS Express stent [19], and thus reduce the number of study subjects, particularly in the control arm. In the PERSEUS WH trial, historical TAXUS Express stent data from the TAXUS IV and TAXUS V trials [1,7] may be borrowed under certain conditions to augment data from the TAXUS Express control group, using subjects who had similar target lesion characteristics (lesion length ≤28 mm, RVD 2.75 mm - 4.0 mm) as those enrolled in the PERSEUS WH study. The observed 12-month TLF rates in these historical cohorts was 8.2% (44/535) for TAXUS IV and 10.9% (33/304) for TAXUS V. Historical control data will only be borrowed if the observed 12-month TLF rate in the TAXUS Express control group enrolled in PERSEUS WH exceeds 8.0%. If the observed TLF rate for PERSEUS WH subjects treated with the TAXUS Express stent is ≤8.0%, historical data will not be borrowed as doing so would raise the TLF rate in TAXUS Express stent subjects in the non-inferiority comparison and potentially bias the analysis in favor of the TAXUS Element stent. This design is therefore more conservative than non-conditional borrowing. The weight of the historical data will depend on how closely the results from the concurrent control match those from the historical controls. If data are borrowed, data from approximately 119 patients (if 12% TLF rate observed) to 199 patients (if 9% TLF rate observed) will be "effectively" borrowed from the historical control, as discussed by Malec et al, 2001 [20]. Based on discussions with the US FDA, a non-inferiority margin (Δ) of 4.1% was chosen and non-inferiority of the TAXUS Element stent will be accepted if the Bayesian posterior probability (θ_1 _- θ_2 _< 0.041 | data) is at least 0.95, where θ_1 _is the 12-month TLF rate for TAXUS Element and θ_2 _is the 12-month TLF rate for TAXUS Express. This non-inferiority margin preserves at least half of the treatment difference observed between TAXUS Express and the lesion-diameter-matched bare metal Express stent control in the combined TAXUS IV and V trials [1,7]. This difference in TLF was deemed to be clinically indistinguishable from a treatment choice perspective and is similar to non-inferiority margins used in other studies comparing DES [21,22]. The sample size of 1264 subjects (which is expected to result in 1200 evaluable subjects assuming 5% attrition) was determined through simulations based on hierarchical modeling. Although power and type I error do not apply to PERSEUS WH in the frequentist sense, this sample size was selected because it was the minimum sample size required to give approximately an 80% probability of correctly concluding non-inferiority (over a range of assumed TAXUS Express TLF rates from 6% to 12%) if the TAXUS Element TLF rate is indeed non-inferior to the TAXUS Express TLF rate.

#### PERSEUS SV

For the PERSEUS SV study, a 2-sided *t*-test will be used to determine if the 9-month in-stent late loss observed for the TAXUS Element stent is superior to that observed for the bare metal Express stent historical control subjects in the TAXUS V trial. The null hypothesis that the true difference in means (TAXUS Element - bare metal Express) is equal to zero will be tested against the two-sided alternative that the true difference in means is different from zero. The sample size was calculated for a two-group test of means using nQuery Advisor^® ^Version 5 (Statistical Solutions Ltd., Saugus, Massachusetts, USA). The expected 9-month in-stent late loss for the TAXUS Element stent is 0.55 mm and the 9-month in-stent late loss for the Express stent is the observed mean (0.77 mm) from the TAXUS V study in subjects with visual estimate RVD ≥2.25 mm to <2.75 mm and lesion length ≤20 mm [7]. The common standard deviation is assumed to be 0.6 mm, which is derived from the TAXUS V Express stent cohort. Given a two-sided α of 0.05, 190 TAXUS Element stent subjects will provide 85% power to reject the null hypothesis if it is indeed false. Further allowance for approximately 15% attrition based on a QCA endpoint resulted in a study enrollment target of 224 subjects.

All frequentist statistical analyses will be done using The SAS System Version 8.2 software or above (SAS Institute Inc., North Carolina, USA). Software for the Bayesian hierarchical modeling was developed by Professor Ming-Hui Chen (University of Connecticut) and was written using the Fortran 90 language and compiled with the Intel Visual Fortran Compiler Professional Edition for Windows with IMSL Version 10.1 or above (Intel Corporation, Santa Clara, California, USA). This software was also used to run simulations of sample size, power and type I error based on the hierarchical models and specialized Gibbs sampling algorithms.

### Study Organization and Ethical Considerations

An independent clinical events committee will adjudicate all reported events of stent thrombosis and MACE. An independent data monitoring committee is responsible for oversight of all reported adverse events and aggregate safety data to monitor for incidence of MACE and other trends that may warrant modification or termination of the trials. PERSEUS study organization and oversight committee membership are listed in the acknowledgements.

The Institutional Review Board or Ethics Committee at each participating center approved the study protocol and all subjects provided written informed consent. The protocols and consent forms were consistent with the International Conference on Harmonisation Guidance for Industry E6 Good Clinical Practice, the Declaration of Helsinki, EN ISO 14155-1 and EN ISO 14155-2, and all local regulations, as appropriate. The PERSEUS study protocols were approved by the US FDA under Investigational Device Exception number G060237.

### Limitations of Study Design

The comparator controls for PERSEUS were chosen based on the commercially available stents at the time of study enrollment. Since that time, a next generation paclitaxel-eluting stent (TAXUS Liberté) and 2 dedicated small vessel paclitaxel-eluting stents (TAXUS Express Atom and TAXUS Liberté Atom) have been US FDA approved. Thus, the PERSEUS comparator groups do not represent the most recently available paclitaxel-eluting stents. Although the formal statistical hypotheses were based on TAXUS Express and Express bare metal stent, the PERSEUS results will need to be interpreted in the context of more recent DES studies.

In addition, the study design includes comparisons to historical controls. In PERSEUS, use of historical data could contribute to bias as a result of differences in patient complexity or patterns of treatment between PERSEUS and historical controls. For PERSEUS WH, data from TAXUS IV and V may be borrowed only if the observed TLF rate in the TAXUS Express concurrent control is similar to the TLF rate in the historical TAXUS Express control. This conditional borrowing results in a more conservative test and also minimizes the likelihood that differences between the concurrent and historical controls will bias the non-inferiority comparison.

## Discussion

The TAXUS Element paclitaxel-eluting stent incorporates a new metal alloy in a novel design, intended to facilitate deliverability and improve radiopacity relative to the earlier generation TAXUS Express^2 ^and TAXUS Liberté stent systems. The safety and efficacy of the TAXUS Element stent are being studied in the PERSEUS clinical trial program, which evaluates the TAXUS Element stent in comparison with either the first generation TAXUS Express stent (WH) or a bare metal Express stent (SV). The PERSEUS WH study employs a novel Bayesian statistical design that uses data from prior TAXUS Express studies to increase power while maintaining acceptable type I error and limiting the number of subjects treated in the study. Enrollment is complete in both studies and primary endpoint data are expected in 2010.

## List of Abbreviations

ACC: American College of Cardiology; AHA: American Heart Association; FDA: US Food and Drug Administration; MACE: Major Adverse Cardiac Events (MI, TVR, Cardiac Death); MI: Myocardial Infarction; PCI: Percutaneous Coronary Intervention; QCA: Quantitative Coronary Angiography; RVD: Reference Vessel Diameter; SCAI: Society for Cardiovascular Angiography and Interventions; SV: Small Vessel (PERSEUS Small Vessel Study); TLF: Target Lesion Failure; TLR: Target Lesion Revascularization; TVR: Target Vessel Revascularization; WH: Workhorse (PERSEUS Workhorse Study).

## Competing interests

The authors wish to disclose the following competing interests: DJA, AB, KDD, JH, AN, and SW are full-time employees of and hold equity in Boston Scientific Corporation. LAC serves on the Advisory Board or Speakers Bureau for Medtronic, Abbott, Boston Scientific, Pfizer, Devax, Inc, and holds equity in Boston Scientific, Medtronic, and Devaxx, Inc. DK received research grants from Boston Scientific and Cordis Corporation, and serves on the Advisory Board for Boston Scientific, Cordis Corporation, Abbott-Vascular, and Medtronic.

## Authors' contributions

DJA, LAC, AB, AN, SW, and DJK participated in the study conception, design, and/or coordination, and revised the manuscript critically for important intellectual content. JEH performed preclinical testing, prepared the preclinical results described in Appendix A, and drafted and reviewed the preclinical section of Appendix A. SW led the development of the statistical analysis design and drafted the statistical sections of the manuscript. KDD participated in the conceptual design of the manuscript, and revised it critically for important intellectual content. All authors have read and approved the final manuscript.

## Appendices

### Appendix A: TAXUS Element Stent Design and Preclinical Testing TAXUS Element Stent Design

**Figure 2:**
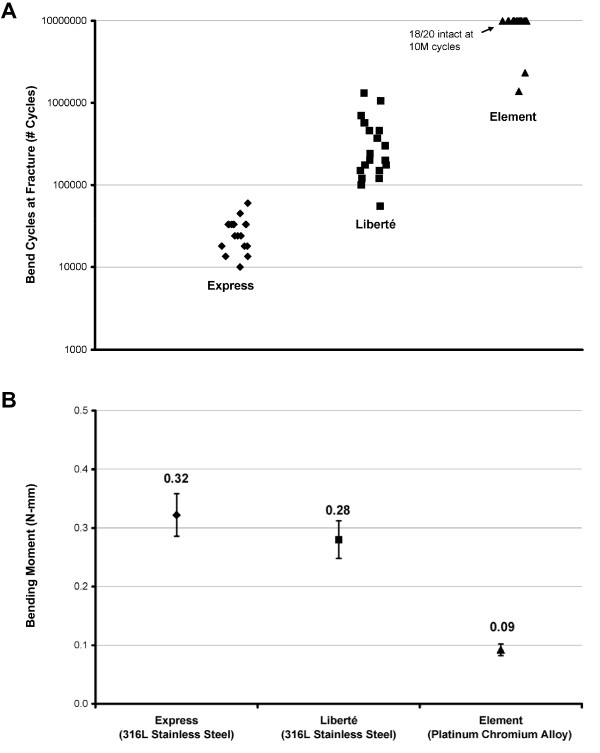
**Strength and flexibility of the TAXUS Element stent compared to TAXUS Express and TAXUS Liberté stents**. (A) Stent integrity, as measured by an accelerated life test of the bending fatigue of a stent in a simulated overlapped stent configuration, showing number of bend cycles before stent fracture. The test is conducted by mounting one end of a nominally deployed stent to a fixed mandrel while the other end is mounted to a mandrel suspended in a flexible membrane. The membrane mounted end of the stent is translated perpendicular to the longitudinal axis of the stent to impart a repeatable bend in the stent. (B) Conformability - a measure of the torque required to bend the stent to a specific curvature, which is directly related to flexibility of the stent. Lower required bending moment indicates increased flexibility. N = 15 for each stent type. Bars represent ± 1 standard deviation.

TAXUS Element incorporates several design changes compared with the TAXUS Express or TAXUS Liberté stent platforms (Table 1). TAXUS Element uses a novel platinum chromium alloy to replace the 316L stainless steel used in previous generation TAXUS stents. This platinum chromium alloy provides increased radial strength and fracture resistance to allow thinner stent struts (Figure 2A). Nominal elemental compositions by weight of the platinum chromium alloy in comparison to other materials are given in Table 2.

**Table 1:** Stent Platform Comparison

Component/Characteristic	TAXUS Express	TAXUS Liberté	TAXUS Element	Impact of Change	
Stent Material	316L Stainless Steel	316L Stainless Steel	Platinum Chromium Alloy	Higher strength & radiopacity	

Drug	Paclitaxel			N/A	

Polymer	Slow-Release Translute polymer			N/A	

Strut Width	71 μm-91 μm	76 μm-94 μm	61 μm-89 μm	Greater flexibility, Lower profile, Reduced inflammation	
	
Strut Thickness*	132 μm	97 μm	81 μm-86 μm		

Nominal Balloon Pressure	9 atm	9 atm for ≤2.50 mm	11 atm	Optimized for stent/balloon configuration	
		8 atm ≥2.75 mm			
	
Balloon Rated Burst Pressure	18 atm (2.25-4.0 mm) 16 atm (4.5-5.0 mm)	18 atm (2.0-4.0 mm) 16 atm (4.5-5.0 mm)	18 atm (2.0-2.25 mm) 16 atm (2.5-5.0 mm)		

Surface-to-Artery Ratio^†^	2.25mm: 20.0%	2.25 mm: 18.6%	2.25 mm: 17.8%	More uniform drug delivery across stent diameters	
	2.50 mm: 18.9%	2.50 mm: 17.6%	2.50 mm: 17.6%		
	3.00 mm: 15.2%	3.00 mm: 19.5%	3.00 mm: 16.4%		
	4.00 mm: 14.6%	4.00 mm: 17.1%	4.00 mm: 15.2%		

* Strut thickness is 86 μm for the 4.00 mm model, and 81 μm for all other models.

† Surface-to-Artery Ratio (SAR) is the percentage of artery wall area covered by outer surface area of the stent. Large values imply a greater surface area of drug coating in direct contact with the vessel and by inference, a larger dose delivered to the local arterial tissue. SAR can vary significantly by diameter for the same stent model. Increasing the number of stent models covering a diameter range decreases the variation of SAR by minimizing the stent's working range.

**Table 2:** Nominal Elemental Composition by Weight (%)

	Platinum Chromium Alloy	316L Stainless Steel	L605 (Cobalt Chromium Alloy)	MP35N (Cobalt Chromium Alloy)
Iron	37*	64*	3.0 max	1.0 max

Platinum	33	-	-	-

Cobalt	-	-	52*	34*

Chromium	18	18	20	20

Nickel	9	14	10	35

Tungsten	-	-	15	-

Molybdenum	2.63	2.63	-	9.75

Manganese	0.05 max	2.00 max	1.50	0.15 max

Titanium	-	-	-	1.0

*Designated as balance; value calculated from nominal values of other elements.

The material properties of platinum chromium, in conjunction with the Element stent design, are expected to provide stent recoil that is similar to 316L stainless steel stent platforms and reduced compared with current cobalt chromium alloy stent platforms. Deployment recoil of the Element stent is 3.6 [95% CI 3.2-4.0] (n = 15) compared to 2.8 [95% CI 2.5-3.1] (n = 25) for the Express stent at a deployment diameter of 3.0 mm, as measured in accordance with ASTM standards [23]. In contrast, deployment recoil of current cobalt chromium stents (Xience and Endeavor) has been measured to be 4.6 [95% CI 4.2-5.0] (n = 10) and 5.0 [95% CI 4.5-5.5] (n = 7), respectively, at a deployment diameter of 3.0 mm.

**Figure 3:**
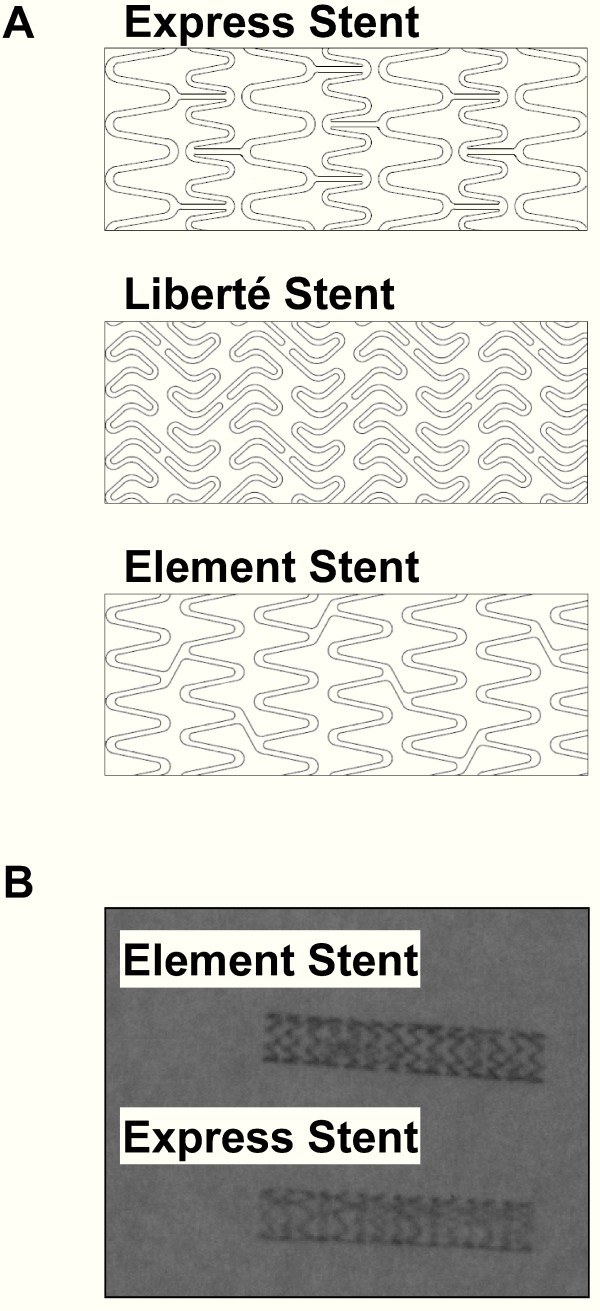
**(A) Express, Liberté, and Element stent architecture; (B) Radiographic comparison of Element and Express stents**. Radiographic image was generated using a General Electric OEC 9800 Digital Imaging System at operating conditions of 51 kV and 11.66 mA. No anatomical simulating phantom was used during imaging.

Several factors correlate with or contribute to radiopacity (x-ray attenuation) of a material, and material density provides a direct relative comparison of stent radiopacity. Density of the platinum chromium alloy (density 9.9 g/cc) is greater than either 316L stainless steel (density 8.0 g/cc) [24] or cobalt chromium (density 8.4 g/cc - 9.1 g/cc, depending on specific stent platform) [24] which should enhance visibility of the thinner struts (Figure 3).

The deliverability of the Element stent may also be improved by changes in stent architecture, including thinner struts as well as fewer connectors between expansion rings (Figure 3). Figure 2B shows a comparison of the ex vivo flexibility of the Element stent compared to the Express and Liberté stents. TAXUS Element is also deployed on a modified Apex balloon catheter delivery system to improve flexibility and reduce balloon withdrawal resistance.

The TAXUS Element stent is coated with styrene-b-isobutylene-b-styrene triblock (SIBS) Translute polymer loaded with paclitaxel (1 μg/mm^2 ^loaded drug/stent surface area). The drug-polymer matrix provides controlled paclitaxel release similar to that of the slow-release TAXUS Express and TAXUS Liberté stents. The continuous cell geometry of the TAXUS Element stent provides more uniform drug delivery along the length of the stent compared to the tandem architecture of the TAXUS Express stent (Figure 3).

### Preclinical Testing

**Figure 4:**
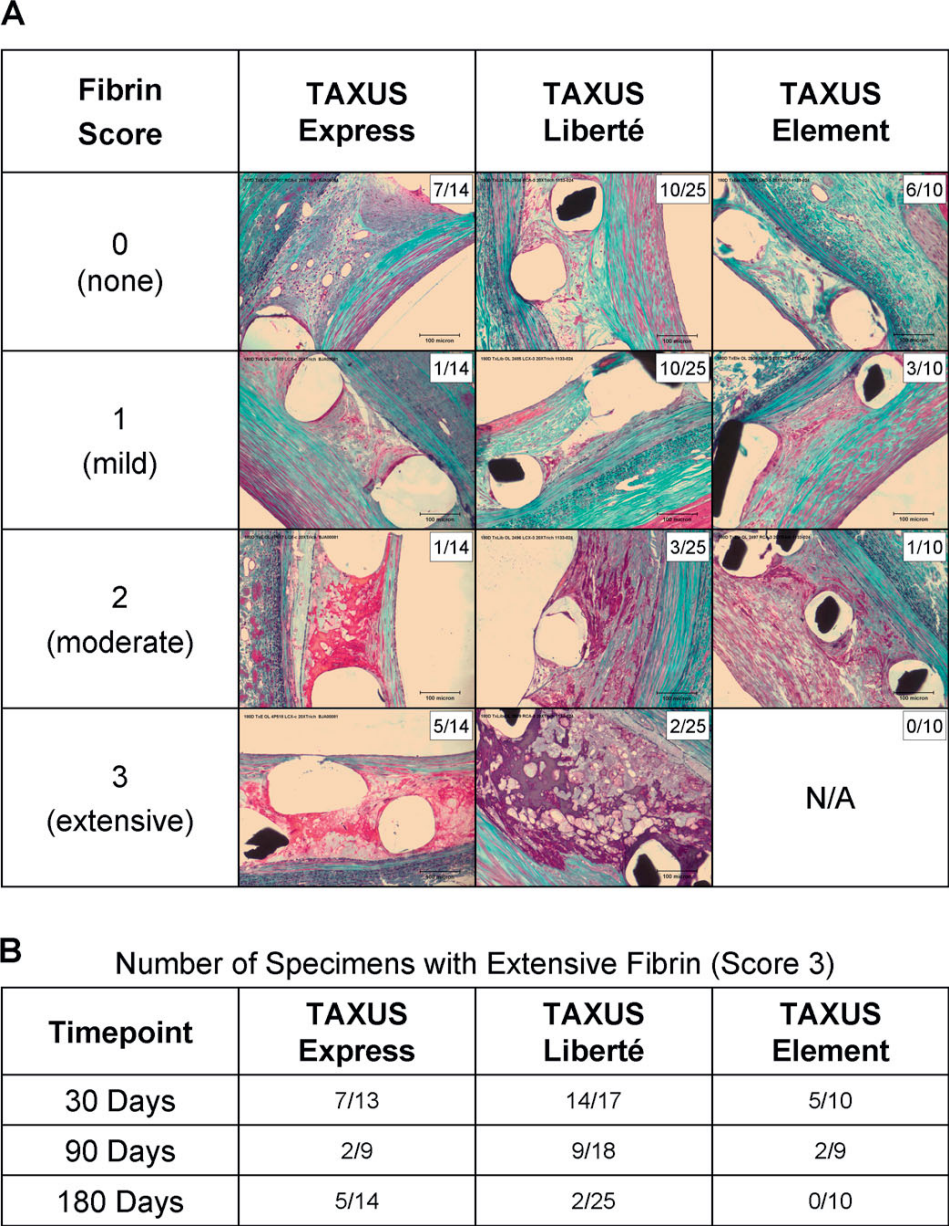
**Fibrin deposition around stent struts following TAXUS stent implantation in porcine coronary arteries**. Swine coronary arteries were implanted with overlapping bare metal or TAXUS Express, TAXUS Liberté, or TAXUS Element paclitaxel-eluting stents and examined at 30, 90, and 180 days using light microscopy. Peristrut fibrin deposition was evaluated by study pathologists and scored on a 0-3 scale where 0 = no visible fibrin, 1 = mild fibrin present, 2 = moderate fibrin present, 3 = extensive fibrin present. Trichrome stained sections, 200× plate magnification. See Seifert et al., 2007 for more detailed methods [29]. (A) Example specimens at 180 days showing peristrut fibrin deposition histology. Number of specimens in each category is shown as n/N. (B) Number of specimens with extensive fibrin deposition (score 3) at each timepoint. There were no significant differences among control bare metal stents in any of the studies.

The normal process of healing following stent-induced injury initially includes the deposition of plasma protein and/or a thrombotic coating of peristrut fibrin containing variable amounts of red blood cells, platelets, and leukocytes [25-27]. It has been suggested that delayed arterial healing following drug-eluting stent implantation is associated with persistent fibrin deposition and reduced or delayed endothelialization, and may be predictive of late stent thrombosis. Stents with thinner struts may be associated with less inflammation and injury to the vessel wall and to become endothelialized more rapidly compared with thicker strut stents [28]. Preclinical studies demonstrate that the thinner-strut Element stent is associated with reduced fibrin deposition and more rapid clearance of fibrin compared with either the TAXUS Express or TAXUS Liberté stents (Figure 4) and suggest that the thin-strut Element stent design may facilitate healing compared to previous generation TAXUS stents.

### Appendix B: PERSEUS WH and PERSEUS SV Inclusion and Exclusion Criteria

#### Clinical Inclusion Criteria

1. Subject is ≥18 years old

2. Eligible for percutaneous coronary intervention (PCI)

3. Documented stable angina pectoris or unstable angina pectoris, or documented silent ischemia

4. Acceptable candidate for coronary artery bypass grafting (CABG)

5. Left ventricular ejection fraction (LVEF) is ≥30%

6. Subject (or legal guardian) understands the study requirements and the treatment procedures and provides written Informed Consent before any study-specific tests or procedures are performed

7. Subject willing to comply with all specified follow-up evaluations

#### Angiographic Inclusion Criteria (Visual Estimate)

1. Target lesion located in native coronary artery

2. Target lesion must be *de novo*

3. Target lesion diameter stenosis ≥50%

4. Reference vessel diameter (RVD):

PERSEUS WH: ≥2.75 mm to ≤4.0 mm

PERSEUS SV: ≥2.25 mm to <2.75 mm

5. Cumulative target lesion length (area to be treated must be completely coverable by one study stent)

PERSEUS WH: ≤28 mm

PERSEUS SV: ≤20 mm

6. Target lesion is successfully pre-dilated. Subjects are enrolled only after successful balloon catheter pre-dilation of the target lesion.

7. One non-target lesion may be treated in a non-target vessel

8. Non-target lesion in non-target vessel must be treated with a commercially available TAXUS stent if use of drug-eluting stent required.

9. Treatment of a non-target lesion (if performed) must be deemed a clinical angiographic success, without requiring use of unplanned additional stent(s).

10. Treatment must be completed prior to treatment of target lesion.

#### Clinical Exclusion Criteria

1. Contraindication to ASA, or to both clopidogrel and ticlopidine

2. Known hypersensitivity to paclitaxel

3. Known allergy to stainless steel

4. Known allergy to platinum

5. Previous treatment of the target vessel with any anti-restenotic drug-coated or drug-eluting coronary stent

6. Previous treatment of the target vessel with a bare metal stent (BMS) within 9 months of the index procedure

7. Previous treatment of any non-target vessel with any anti-restenotic drug-coated or drug-eluting coronary stent within 9 months of the index procedure

8. Previous treatment with intravascular brachytherapy in the target vessel

9. Planned PCI or CABG post-index procedure

10. Planned or actual target vessel treatment with an unapproved device, directional or rotational coronary atherectomy, laser, cutting balloon or transluminal extraction catheter immediately prior to stent placement

11. Myocardial infarction (MI) within 72 hours prior to the index procedure as defined per protocol definition (see Appendix B)

12. Cerebrovascular accident (CVA) within the past 6 months

13. Cardiogenic shock characterized by systolic pressure < 80 mm Hg and/or central filling pressure > 20 mm Hg, or cardiac index < 1.8 liters/minute/m2 or intra-aortic balloon pump or intravenous inotropes are needed to maintain a systolic pressure > 80 mm Hg and a cardiac index > 1.8 liters/minute/m2

14. Acute or chronic renal dysfunction (creatinine > 2.0 mg/dl or 177 μmol/l)

15. Contraindication to ASA, or to both clopidogrel and ticlopidine

16. Known hypersensitivity to paclitaxel

17. Known allergy to stainless steel

18. Known allergy to platinum

19. Previous treatment of the target vessel with any anti-restenotic drug-coated or drug-eluting coronary stent

20. Previous treatment of the target vessel with a bare metal stent (BMS) within 9 months of the index procedure

21. Previous treatment of any non-target vessel with any anti-restenotic drug-coated or drug-eluting coronary stent within 9 months of the index procedure

22. Previous treatment with intravascular brachytherapy in the target vessel

23. Planned PCI or CABG post-index procedure

24. Planned or actual target vessel treatment with an unapproved device, directional or rotational coronary atherectomy, laser, cutting balloon or transluminal extraction catheter immediately prior to stent placement

25. Myocardial infarction (MI) within 72 hours prior to the index procedure as defined per protocol definition (see Appendix B)

26. Cerebrovascular accident (CVA) within the past 6 months

27. Cardiogenic shock characterized by systolic pressure < 80 mm Hg and/or central filling pressure > 20 mm Hg, or cardiac index < 1.8 liters/minute/m2 or intra-aortic balloon pump or intravenous inotropes are needed to maintain a systolic pressure > 80 mm Hg and a cardiac index > 1.8 liters/minute/m2

28. Acute or chronic renal dysfunction (creatinine > 2.0 mg/dl or 177 μmol/l)

29. Any prior true anaphylactic reaction to contrast agents; defined as known anaphylactoid or other non-anaphylactic allergic reactions to contrast agents that cannot be adequately pre-medicated prior to the index procedure

30. Leukopenia (leukocyte count < 3.5 × 109/liter)

31. Thrombocytopenia (platelet count < 100,000/mm3)

32. Thrombocytosis (> 750,000/mm3)

33. Active peptic ulcer or active gastrointestinal (GI) bleeding

34. Current treatment, or past treatment within 12 months of the index procedure, with paclitaxel or other chemotherapeutic agent(s)

35. Anticipated treatment with paclitaxel or oral rapamycin during any period in the 9 months after the index procedure

36. Male or female with known intention to procreate within 9 months after the index procedure

37. Positive pregnancy test within 7 days before the index procedure, or lactating

38. Life expectancy of less than 24 months due to other medical conditions

39. Co-morbid condition(s) that could limit the subject's ability to comply with study follow-up requirements or impact the scientific integrity of the study

40. Currently participating in another investigational drug or device study

41. Any prior true anaphylactic reaction to contrast agents; defined as known anaphylactoid or other non-anaphylactic allergic reactions to contrast agents that cannot be adequately pre-medicated prior to the index procedure

42. Leukopenia (leukocyte count < 3.5 × 109/liter)

43. Thrombocytopenia (platelet count < 100,000/mm3)

44. Thrombocytosis (> 750,000/mm3)

45. Active peptic ulcer or active gastrointestinal (GI) bleeding

46. Current treatment, or past treatment within 12 months of the index procedure, with paclitaxel or other chemotherapeutic agent(s)

47. Anticipated treatment with paclitaxel or oral rapamycin during any period in the 9 months after the index procedure

48. Male or female with known intention to procreate within 9 months after the index procedure

49. Positive pregnancy test within 7 days before the index procedure, or lactating

50. Life expectancy of less than 24 months due to other medical conditions

51. Co-morbid condition(s) that could limit the subject's ability to comply with study follow-up requirements or impact the scientific integrity of the study

52. Currently participating in another investigational drug or device study

53. Any prior true anaphylactic reaction to contrast agents; defined as known anaphylactoid or other non-anaphylactic allergic reactions to contrast agents that cannot be adequately pre-medicated prior to the index procedure

54. Leukopenia (leukocyte count < 3.5 × 109/liter)

55. Thrombocytopenia (platelet count < 100,000/mm3)

56. Thrombocytosis (> 750,000/mm3)

57. Active peptic ulcer or active gastrointestinal (GI) bleeding

58. Current treatment, or past treatment within 12 months of the index procedure, with paclitaxel or other chemotherapeutic agent(s)

59. Anticipated treatment with paclitaxel or oral rapamycin during any period in the 9 months after the index procedure

60. Male or female with known intention to procreate within 9 months after the index procedure

61. Positive pregnancy test within 7 days before the index procedure, or lactating

62. Life expectancy of less than 24 months due to other medical conditions

63. Co-morbid condition(s) that could limit the subject's ability to comply with study follow-up requirements or impact the scientific integrity of the study

64. Currently participating in another investigational drug or device study

#### Angiographic Exclusion Criteria (Visual Estimate)

1. Target lesion in left main artery, whether protected or unprotected

2. Target lesion is a chronic total occlusion (TIMI flow < 1)

3. Target lesion is restenotic

4. Target lesion is located in a saphenous vein graft or mammary artery graft

5. Target lesion is accessed via saphenous vein graft or mammary artery graft

6. Target lesion is < 5 mm from bare metal stent (BMS)

7. Target lesion is < 5 mm from ostium

8. Target lesion is < 5 mm from a side branch vessel ≥ 2.0 mm in diameter (Exceptions: subject may be enrolled if side branch is 100% occluded or if side branch is protected with a patent graft)

9. Untreated lesions with ≥ 50% diameter stenosis or thought to impair flow remaining in target vessel at a location with ≥ 2.0 mm RVD

10. Target lesion and/or target vessel proximal to the target lesion is moderately or severely calcified

11. Target lesion and/or target vessel proximal to the target lesion is severely tortuous

12. Target lesion is located within or distal to a > 60° bend in the vessel

13. Target lesion with angiographic presence of probable or definite thrombus

14. Unprotected left main coronary artery disease

15. Protected left main coronary artery disease with target lesion in LAD or LCx (subject may be enrolled if only lesion is target lesion in RCA)

### Appendix C: PERSEUS WH and PERSEUS SV Definitions

#### Binary Restenosis

Diameter stenosis >50% at the previously treated lesion site, including the original treated area and adjacent proximal and distal QCA analysis segment (see the Angiographic Core Laboratory Manual within the Site Manual of Operations).

#### Clinical Angiographic Success for Non-Target Lesion

Mean lesion diameter stenosis <50% (<30% for stents) in 2 near-orthogonal projections with TIMI 3 flow, as visually assessed by the physician, without the occurrence of prolonged chest pain or ECG changes consistent with myocardial infarction.

#### Clinical Procedural Success (Visual Estimate)

Mean lesion diameter stenosis <30% in 2 near-orthogonal projections with TIMI 3 flow, as visually assessed by the physician, without the occurrence of in-hospital MACE.

#### Death

Death is divided into 2 categories:

*Cardiac death *is defined as death due to any of the following:

1. Acute myocardial infarction

2. Cardiac perforation/pericardial tamponade

3. Arrhythmia or conduction abnormality

4. Cerebrovascular accident through hospital discharge or cerebrovascular accident suspected of being related to the procedure

5. Death due to complication of the procedure, including bleeding, vascular repair, transfusion reaction, or bypass surgery

6. Any death in which a cardiac cause cannot be excluded

*Non-cardiac death *is defined as a death not due to cardiac causes (as defined above).

#### % Diameter Stenosis

Angiographic % diameter stenosis (% DS) was defined as (1-[MLD/RVD])×100.

#### Late Loss

Post-procedure MLD minus follow-up MLD as determined by quantitative angiography.

#### Major Adverse Cardiac Events (MACE)

An event of MI and/or an event resulting in TVR and/or cardiac death are considered MACE events for this study.

#### Myocardial Infarction

Myocardial Infarction will be defined as either:

1. Q-wave MI: Development of new (i.e., not present on the subject's ECG before allocation) pathological Q-waves in 2 or more leads lasting ≥ 0.04 seconds with post procedure CK-MB levels elevated above normal.

2. Non-Q-Wave MI: De novo elevation of CK Total levels > 2.0 × ULN without the presence of new Q-waves (not present on the subject's ECG before allocation). If CK-MB performed, it must be positive.

For subjects undergoing bypass surgery, a perioperative MI will be defined as (a) Total CK-MB > 5× upper limits of local laboratory normal, or (b) Presence of new pathologic Q waves (as defined above).

#### Stent Thrombosis

*NOTE: Data will be collected which will allow for reporting per Boston Scientific's Historical (TAXUS IV and V) stent thrombosis definition as well as the Academic Research Consortium stent thrombosis definition *[12].

*Boston Scientific Historical (TAXUS IV and V *[1,7]) *Stent Thrombosis Definition*:

The occurrence of any of the following:

1. Clinical presentation of acute coronary syndrome with angiographic evidence of stent thrombosis:

• Angiographic documentation of acute complete occlusion (TIMI flow 0 or 1) of the treated area in a previously successfully treated artery (TIMI flow 2 to 3 immediately after stent placement and diameter stenosis ≤ 30%) and/or

• Angiographic documentation of a flow limiting thrombus within or adjacent to the successfully treated lesion.

2. Acute MI in the distribution of the treated vessel.

3. Cardiac death within the first 30 days post index procedure (without other obvious cause) is considered a surrogate for stent thrombosis when angiography is not available.

Stent thrombosis will be classified as follows:

1. "Confirmed stent thrombosis" for the description of above events with angiographic evidence.

2. "Presumed stent thrombosis" for the description of above events in the absence of an angiography (i.e., such as in the case of death without autopsy).

*Academic Research Consortium Definition*:

Stent thrombosis will also be defined per the Definite, Probable, and Possible definitions described in Cutlip et al., 2007 [12].

*Timing*:

Acute stent thrombosis:   0 - 24 hours post stent implantation

Subacute stent thrombosis:   >24 hours - 30 days post stent implantation

Late stent thrombosis:   >30 days - 1 year post stent implantation

Very late stent thrombosis:   >1 year post stent implantation

#### Target Lesion Failure (TLF)

Any ischemia-driven revascularization of the target lesion (TLR), MI (Q-wave and non-Q-wave) related to the target vessel, or (cardiac) death related to the target vessel.

For the purposes of this protocol, if it cannot be determined with certainty whether MI or death was related to the target vessel, it will be considered TLF.

#### Target Lesion Revascularization (TLR)

Target Lesion Revascularization is defined as any ischemia-driven repeat percutaneous intervention (to improve blood flow) of the successfully treated target lesion or bypass surgery of the target vessel with a graft distally to the successfully treated target lesion.

A target lesion revascularization will be considered as ischemia-driven if the target lesion diameter stenosis is ≥ 50% by QCA and there is presence of clinical or functional ischemia which cannot be explained by other coronary or graft lesions. Clinical or functional ischemia is any of the following:

1. The subject has a positive functional study corresponding to the area served by the target lesion

2. The subject has ischemic ECG changes at rest in a distribution consistent with the target vessel

3. The subject has ischemic symptoms referable to the target lesion.

A target lesion revascularization will be considered as ischemia-driven if the lesion diameter stenosis is ≥ 70% by QCA *even *in the absence of clinical or functional ischemia.

#### Target Vessel Failure (TVF)

Any ischemia-driven revascularization of the target vessel, MI (Q- and non-Q-wave) related to the target vessel, or death related to the target vessel.

For the purposes of this protocol, if it cannot be determined with certainty whether MI or death was related to the target vessel, it will be considered TVF.

#### Target Vessel Revascularization (TVR)

Presence of any Target Lesion Revascularization or Target Vessel Revascularization Remote.

#### Target Vessel Revascularization, Remote (TVR, Non-TLR)

Target Vessel Revascularization, Non-TLR is defined as any ischemia-driven repeat percutaneous intervention (to improve blood flow) or bypass surgery of not previously existing lesions ≥ 50% by QCA in the target vessel, excluding the target lesion.

A target vessel revascularization will be considered ischemia-driven if the target vessel diameter stenosis is ≥ 50% by QCA and any of the following are present:

1. The subject has a positive functional study corresponding to the area served by the target vessel

2. The subject has ischemic ECG changes at rest in a distribution consistent with the target vessel

3. The subject has ischemic symptoms referable to the target vessel.

A target vessel revascularization will also be considered as ischemia-driven if the lesion diameter stenosis is ≥ 70% *even *in the absence of clinical or functional ischemia.

#### Technical Success

Technical success is defined as successful delivery and deployment of the study stent to the target vessel, without balloon rupture or stent embolization.
